# Which treatment is most effective at reducing the damage caused by the pest box tree moth? A case in a Mediterranean mountain

**DOI:** 10.7717/peerj.21800

**Published:** 2026-10-22

**Authors:** Fiona Corominas, Lluís Vilar, Jordi Bou

**Affiliations:** GRECO, Institute of Aquatic Ecology, Universitat de Girona, Girona, Catalonia, Spain

**Keywords:** Field trial, Boxwood, *Buxus sempervirens*, *Cydalima perspectalis*, *Bacillus thuringiensis*, *Trichogramma* spp., Mass capture

## Abstract

Boxwood (*Buxus sempervirens*) is an important element of the European plant landscape. Currently, this species is seriously threatened due to the high impact of the pest box tree moth (*Cydalima perspectalis*). This lepidopteran is an invasive species of Asian origin that feeds exclusively on the genus *Buxus*. Management of this pest is necessary, but field trials are lacking. The aim of this study is to provide robust information about the effectiveness of the three major control methods currently available through a case study on a Mediterranean mountain. These treatments include the application of *Bacillus thuringiensis* and mass capture with sex pheromones in combination with the use of parasitoids of the genus *Trichogramma*. The experiment was carried out at Pla Boixer, NE Iberian Peninsula. Between 2020 and 2022, damage to boxwood was sampled and analysed at different scales. The results show that *Bacillus thuringiensis* reduced defoliation by 53%, whereas mass trapping only preserved regrowth with no significant reduction in damage (*p* > 0.05). *Trichogramma* releases did not significantly affect damage levels, nor in combination with other treatments. The treatment with *B. thuringiensis* protected the boxwood from defoliation but its use should be limited to specific cases in which boxwood is highly affected at the regional scale because of its impact on ecosystems and the difficulty of its application. No seasonality was observed in the damage; however, regrowth showed a favourable trend from spring to autumn. These insights should be considered in future management plans for this pest.

## Introduction

### The boxwood

Boxwood (*Buxus sempervirens* Linnaeus, 1753) is an evergreen shrub of the Buxaceae family that is monoecious and slow growing. It is a common forest species in Europe that shows a centre of continuity, abundance and frequency in the Pyrenees, southern France, French Prealps and all around the Jura Mts (Di Domenico, Lucchese & Magri, 2012). It is also widely distributed in Black Sea-facing slopes of Turkey and also has significant distribution areas in the Mediterranean region (Sarı, Çelikel & Yaşar, 2023). Fragmented populations are found in southern Europe and in central France, where the climate is Mediterranean. Boxwood primarily grows in limestone soils but occasionally also in siliceous soils, as observed in Belgium (Duvigneaud, 1969). It can be found at elevations between sea level and 2,000 m under subhumid conditions on long slopes, river valleys, canyons, gorges, ravines, and thermal springs (Di Domenico, Lucchese & Magri, 2012). Boxwood is a crucial element in European landscapes and is a very abundant species in the shrub layer of various deciduous forests. In Mediterranean calcareous mountains, boxwood woodlands constitute a habitat of community interest (Natura 2000 code 5110).

Boxwood formations create dense thickets, generating microenvironments that favour biodiversity closely linked to this species. In a study by Mitchell et al. (2018), various databases were reviewed, revealing that at least 43 species of fungi, three of chromists (algae), and 18 of invertebrates are specific to *Buxus* spp. Additionally, boxwood is crucial for maintaining soil stability and water quality in its environment because its sediment capture capacity on steep slopes provides substrate fixation (Duvigneaud, 1969; Mitchell et al., 2018).

Likewise, boxwood holds major sociocultural importance due to its traditional and current uses. Its wood has been used since ancient times to craft tools and utensils because it is one of the heaviest and hardest woods among all European trees (Savill, 2013). Moreover, in ancient times, the plant was used as a treatment for malaria and intestinal infections primarily because of its high concentration of alkaloids (Leuthardt, Glauser & Baur, 2013). It is also widely used as an ornamental plant in, with subspecies being imported from other regions due to the high commercial demand (Mitchell et al., 2018; Suppo, Bras & Robinet, 2020). The intensification of international trade in ornamental plants worldwide during the second half of the 20th century has led to a significant increase in the number of exotic phytophagous insects associated with these plants (Roques, 2010; Suppo, Bras & Robinet, 2020).

### The box tree moth

The box tree moth *Cydalima perspectalis* (Walker, 1859) is a species of nocturnal moth (Lepidoptera: Crambidae). This species is native to temperate and subtropical regions of East Asia, including China, Japan, and Korea (Inoue, 1982). Currently, it is an invasive pest in various parts of Europe, where it continues to spread. Its impact on natural ecosystems is substantial, causing defoliation of boxwood (Nacambo et al., 2014).

Its presence in Europe was first recorded in 2007 in southwestern Germany (Krüger, 2008). This introduction was considered accidental and attributed to the trade and transport of ornamental plants of the genus *Buxus* (Krüger, 2008; Muus, Van Haaften & Van Deventer, 2009). It then spread to most European countries with the presence of *Buxus* spp. and optimal climatic conditions for moth development (Kenis et al., 2013; Mitchell et al., 2018; Bras et al., 2019). The first record of *Cydalima perspectalis* on the NE Iberian Peninsula was recorded in 2014 in the Garrotxa region (Girona’s province) (Bassols & Oliveras, 2014).

The distribution of this invasive species is closely tied to the presence of boxwood because its larvae feed exclusively on the leaves of *Buxus* spp. and occasionally on the bark. Current studies rule out other potential hosts, making this process solely linked to plants of the genus *Buxus* (Van der Straten & Muus, 2010; Wan et al., 2014). Therefore, its life cycle is closely linked to this plant (Nacambo et al., 2014; Wan et al., 2014). The larvae feed on boxwood until they reach maturity and moths fly over boxwood populations to initiate their cycle again (Van der Straten & Muus, 2010; Leuthardt & Baur, 2013).

The extent of this moth’s expansion may still increase in the near future. The potential distribution of this moth indicates a successful establishment in most of Europe, excluding most regions in northern Europe (Nacambo et al., 2014). However, populations and damage are likely to be greater in the southern half of Europe, where *C. perspectalis* can develop two or more generations per year (Nacambo et al., 2014; Matošević et al., 2017). The optimal climatic conditions for the development of *C. perspectalis* are mild temperatures with low seasonal precipitation combined with a narrow temperature range during the day (Suppo, Bras & Robinet, 2020).

As an insect, *C. perspectalis* is a poikilothermic organism, and one of the factors that most influences its development and activity is temperature, along with relative humidity and photoperiod (Maruyama & Shinkaji, 1987). Larval development is faster with higher temperatures and longer photoperiods (Maruyama & Shinkaji, 1991). For this reason, the increase in temperatures due to global change can lead to a reduction in development time, expansion towards northern latitudes, and a delay in diapause in the fall, favouring the completion of an additional generation (López & Eizaguirre, 2019).

The main damage caused by the box tree moth *Cydalima perspectalis* is the complete defoliation of boxwood, which can lead to the death of individuals and severely affect natural populations of *Buxus* spp. (Kenis et al., 2013; Matošević, 2013; Nacambo et al., 2014). This ecological impact becomes particularly serious when it comes to the main areas of natural distribution of *B. sempervirens* in Europe, as boxwood is an essential element of the natural habitats of the region, such as the southern part of the Massif Central in France and the Pyrenees. These damages have serious consequences not only for boxwood but also for the overall functioning of the unique forest ecosystems (Di Domenico, Lucchese & Magri, 2012; Kenis et al., 2013; Nacambo et al., 2014). Currently, *C. perspectalis* infestations have been observed in large forested areas with boxwood, as seen in Switzerland (Leuthardt & Baur, 2013) or in the Russian Caucasus (Gninenko & Sergeeva, 2018). The expansion of *C. perspectalis* has been observed in the Mediterranean Mountains, such as in the NE Iberian Peninsula, where the infestation has reached the Ebro lands. It is also expanding to the west of Catalonia, where the availability of food has drastically decreased in the most affected inland regions, leading to a high percentage of heavily defoliated boxwoods (Observatori Forestal Català, 2022). There are areas where the infestation has not yet occurred, such as lands that are higher than the current altitudinal limit of *C. perspectalis*, which varies depending on the environmental conditions of each region and which could be affected by rising temperatures (López & Eizaguirre, 2019; Canelles et al., 2021).

Although this is a widespread issue, it is currently unknown how these ecosystems respond to the rapid death of large boxwood populations. However, an event with such a high degree of influence on its environment can significantly impact soil balance. Additionally, in areas with high erosion, the soil may be impoverished since the new vegetation will not grow fast enough to stabilize the slope and maintain the protective function of the communities growing on these slopes (CABI, 2021).

The great damage it causes in areas where it is an invasive species is thought to be related to the absence of natural predators of the species in these European zones (Wan et al., 2014). This absence of predators is partly because the genus *Buxus* accumulates toxic steroidal alkaloids in its leaves, roots, and stems. Consequently, the fact that *C. perspectalis* is a specific monophagous species means that it accumulates a significant amount of these alkaloids, reaching concentrations up to 20 times greater than those found in the leaves (Leuthardt, Glauser & Baur, 2013). This makes the larvae toxic to birds and other potential predator species. Although individuals of some bird and insect species have been occasionally observed eating *C. perspectalis* larvae, this occurrence is currently anecdotal and does not have a significant impact on reducing the pest.

### Control methods

Due to the invasive nature of this insect and its devastating ecological impacts, efficient pest control is needed. The main control methods used are phytosanitary treatment with *Bacillus thuringiensis*, male-attracting pheromones for mass trapping, and the use of commercially available parasitoids such as *Trichogramma* spp. There are studies on these treatments, but field trials and management experience are lacking. There are also other control methods being tested in Europe (Korycinska & Eyre, 2011; Rose et al., 2013; Wan et al., 2014; Göttig, 2017; Tabone et al., 2023), but they are in more experimental stages with few case studies. However, there is a lack of field trails to evaluate the effectivity on boxwood populations at natural areas and comparations between treatments.

The phytosanitary treatment with *Bacillus thuringiensis* var. *kurstaki* (Btk) (Berliner, 1915) uses entomopathogenic bacteria as a biological control that only affects lepidopteran larvae. This treatment does not have short-term effects on non-target diurnal lepidopterans’ abundance or species richness (Barbero et al., 2024). This degree of specificity makes it one of the most widely used biological pesticides, with a high level of efficiency and success in controlling *C. perspectalis* larvae (Cawoy et al., 2011; Lacey et al., 2015). The larvae ingest the bacteria, inducing intestinal and haemolymph poisoning, leading to insect death after a few days (Burges & Hurst, 1977). However, a problem arises if it is applied as the sole biological control measure, as there is a risk of developing resistance when it is applied recurrently (Schnepf et al., 1998). Its application is carried out by spraying a phytosanitary product on boxwood bushes (Senmartí & Las Heras, 2020).

Mass trapping using male-attracting pheromone traps is a semichemical control method that affects the adult stage, specifically of males, and influences the reproductive capacity of the species. Initially, Kawazu et al. (2007) analysed the sexual pheromones of *C. perspectalis* populations in Japan and concluded that they could be used for species control. However, tests with the Japanese pheromone in Europe have not been satisfactory (Van der Straten & Muus, 2010), possibly because of a difference in sensitivity in the European population (Pherobank, 2018). For this reason, specific pheromones have been developed for European moth populations (Pherobank, 2018). The effectiveness of this method in capturing adults is high (Burjanadze, Supatashvili & Gorkturk, 2019), but it seems to be less effective in terms of its pest control action (Askari, Sefidkon & Allahverdi Mamaghani, 2023).

*Trichogramma* spp. is a biological control agent that consists of a mixture of parasitoids from the *Trichogramma* genus, which are natural enemies of *C. perspectalis* in its place of origin. These parasitic wasps lay their eggs on the eggs of their hosts, killing them (Stouthamer & Kazmer, 1994). Parasitism by this genus has a low acceptance rate by users. However, *T. dendrolimi* stands out as the most effective species according to some *ex situ* studies (Göttig & Herz, 2016a; Göttig, 2017), although its searching ability is low (Göttig, 2017), which poses a problem if it is intended to be used as a control method for the moth. The effectiveness of *Trichogramma* spp. under field conditions remains unknown (Wan et al., 2014).

Due to the lack of field trials with these three major pest control treatments (*Bacillus thuringiensis*, mass trapping using pheromone traps and parasitoids of the genus *Trichogramma*), more insights are needed about the pest management in natural areas. Based on the current knowledge, the hypothesis are: (1) treatment with *B. thuringiensis* is the most effective control method and that (2) mass trapping is less effective; (3) the use of *Trichogramma* parasitoids effectiveness is not expected in the natural environment. Consequently, the main objective of this study was to employ an evidence-based experimental approach to provide robust information about these treatments against the box tree moth in a Mediterranean mountain. Specifically objectives are: (1) analyse the effectiveness of the treatments in preventing and reducing the degree of damage to natural boxwoods; (2) generate informed recommendations for pest management and conservation of boxwoods and habitats with boxwood presence; (3) consider the biological cycles of the box tree moth and the plant itself, which is why seasonal variations in the parameters of infestation need to be analysed.

## Materials & Methods

### Field trial site selection

The experimental plots are located at the Pla Boixer project (Fig. 1), NE Iberian Peninsula, on the cliffs of Tavertet, in the Osona region. This area is included in the Natura 2000 network within the Catalan Transversal System and the protected area of Collsacabra PEIN. This region, with a Mediterranean climate, consists of distinct cliffs ranging from 900 to 1,000 m above sea level, primarily formed by limestone and sandstone materials (Soler, Pallí & Brusi, 2013).

**Figure 1:**
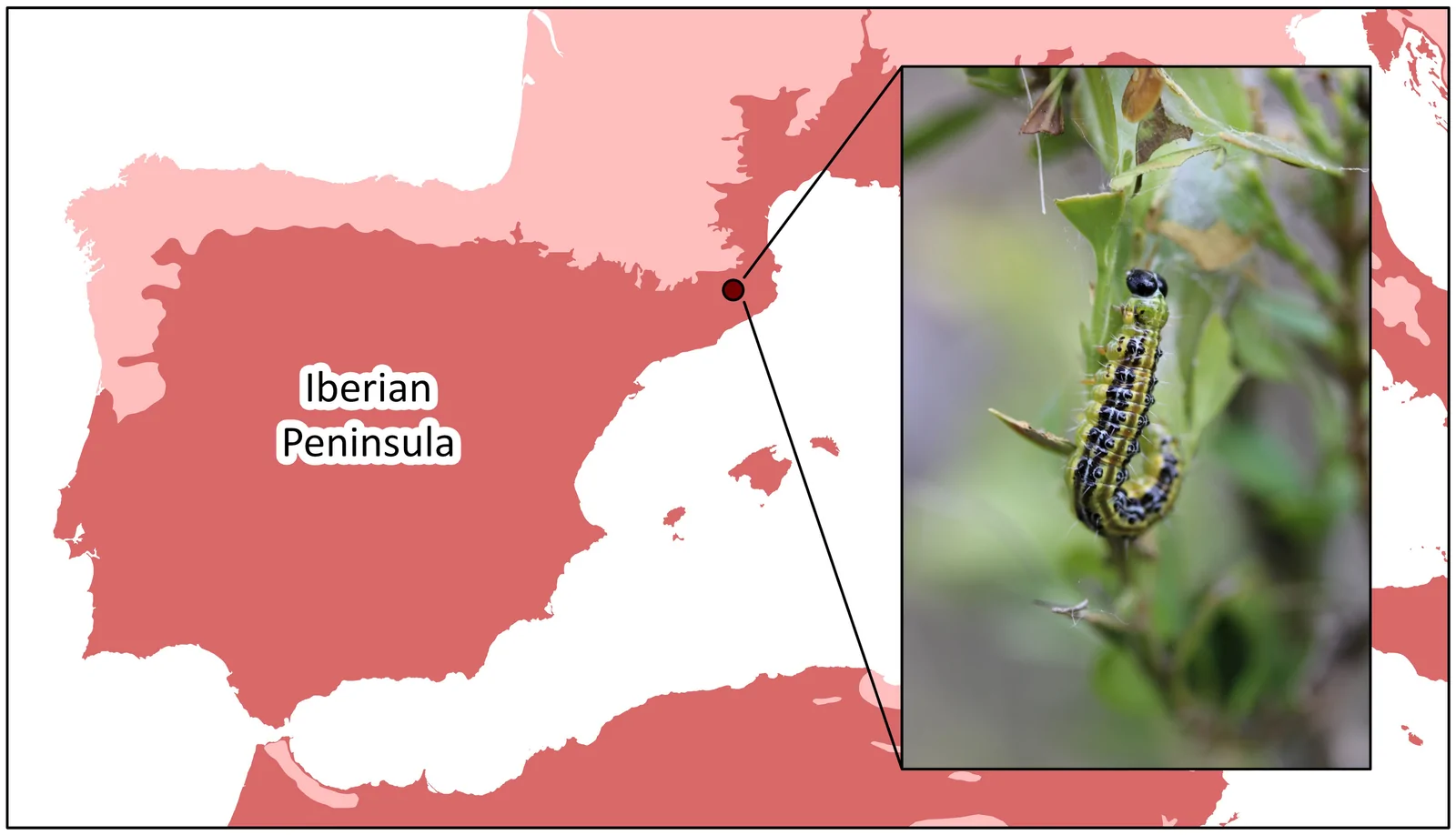
Location of study site (Pla Boixer) in the Iberian Peninsula and *Cydalima perspectalis* larvae eating *Buxus sempervirens*. The mediterranean ecoregion is depicted in dark red.

Specifically, Pla Boixer is situated on one of the cliffs of Tavertet facing south and has a known slope. The experimental plots are located at altitudes between 1,050 and 1,100 m above sea level. In terms of geology, Pla Boixer is composed of limestones with nummulites and sandy limestones from the Lower Lutetian, Cenozoic (ICGC, 2007). The climate in Collsacabra is Mediterranean; at Pla Boixer, the total annual precipitation is 1,129.5 mm, the annual average temperature is 9.8 °C, the annual average maximum temperature is 14.8 °C, and the annual average minimum temperature is 4.9 °C (UAB, 2004). The shrubland is dominated by *B. sempervirens* (94%), with a few other shrubs like *Genista scorpius*, and only isolated trees as holm oaks (*Quercus ilex*) and oaks (*Quercus pubescens*). This vegetation formation corresponds to the 32.641^+^ CORINE habitat.

The current affected area has reached 49% of the territory, with the surface area of affected boxwood estimated at 169,463 hectares (Carballal & Sangermán, 2021). In June 2018, the presence of *C. perspectalis* in Pla Boixer was observed for the first time with homogeneous effects around the area. After detection, treatments against the pest box tree moth began in 2019, and the treatment zones were distributed in Pla Boixer the same year. The treatments are phytosanitary treatment with *Bacillus thuringiensis* var. *kurstaki*, mass capture with sexual pheromones, and the use of *Trichogramma* spp. parasitoids.

### Experimental design

To carry out the study, treatments for the control of *C. perspectalis* were applied to the experimental plots at Pla Boixer. Two factors were considered, Treatment 1 (T1) and Treatment 2 (T2), as shown in Table 1. Treatment 1 is based on the application of the most commonly used methods in pest management (*Bacillus thuringiensis* and mass capture with pheromones) and a control zone (without treatments). For Treatment 2, the introduction of *Trichogramma* spp. parasitoids that parasitize box tree moth eggs is a less investigated technique applied as a complement to Treatment 1. Plots containing *Trichogramma* and plots without *Trichogramma* (corresponding to the T2 control) were established to determine the effectiveness of this method combined with other methods.

**Table 1:** Treatments applied to the experimental plots of Pla Boixer. The two treatment factors are displayed: the most common phytosanitary treatments (T1) and the use of parasitoids (T2), along with their corresponding codes and the number of replicates.

**Treatments**		**Code**	**N° replicates**
**T1**	**T2**		
*Bacillus thuringiensis*	No *Trichogramma*	Bt_nt	4
	*Trichogramma*	Bt_t	4
Control	No *Trichogramma*	C_nt	4
	*Trichogramma*	C_t	4
Mass capture	No *Trichogramma*	F_nt	4
	*Trichogramma*	F_t	4

Consequently, the experimental design stablished three zones for the treatment T1: a control zone (one ha), a *Bacillus thuringiensis* zone (0.9 ha), and a pheromone zone (1.2 ha). These zones were separated by 100–150 m of mountain grasslands and trails to minimize interference among treatments. Within each zone, a 0.5 ha *Trichogramma* subzone was established, constituting the T2 treatment. Four replicates were conducted for each combination of treatments (T1 and T2), resulting in a total of 24 plots. These experimental plots were established within the zones and subzones as blocks, while avoiding edge areas to reduce margin effects.

Specifically, in the *B. thuringiensis* treatment area, the product was sprayed using a cannon and pistol, both by vehicle and manually, in spring (April–May) and summer (July–September). The dose used was 0.25–0.5 kg/ha with a concentration of 32 million of CLU/g. In the pheromone treatment area, a network of male-capture Funnel traps was established at boxwood branches (height of one m approximately), at a density of 100 traps per hectare. In the subzones with *Trichogramma* spp. parasitoid treatment, two releases were carried out, separated by 15 days, using commercial *Tricholine^®^* diffusers (containing *Trichogramma achaeae*) at a dose of one diffuser per 10 m^2^, in July and September (Senmartí & Las Heras, 2020).

### Ecological monitoring

The study was conducted over three years of sampling, from 2020 to 2022 (them included), with two annual sampling campaigns: “Campaign A” in early spring, before the density of young larvae becomes substantial, and “Campaign B” in autumn, when the presence of larvae is no longer observed. Twenty-four square plots of 25 m^2^ each (5 m × 5 m) were established, marked and georeferenced. Monitoring involved assessing the experimental plots at different scales: studying the phytosanitary status of boxwood by considering the degree of defoliation of these plants and performing boxwood population studies. To analyse the effectiveness of the treatments, different variables indicating the condition of the boxwood were used based on the different types of sampling units: plot, individual plant, branch, and new growth (Table S10).

At the plot level, the cover percent and degree of defoliation (%) of boxwood were measured to assess the structure and phytosanitary status of the population. Boxwood was also studied at the population level, with measurements taken on the individual plants, branches, and new growths. Within each plot, eight individual boxwood plants were randomly selected. Sampling of the individual plants involved assessing the overall phytosanitary condition of the plant, the presence of pathogenic fungi, and the percentage of damage caused by defoliation. Additionally, measurements of the branches and new growth of each sampled individual plant were taken. Five branches per plant were randomly selected. Measurements were taken on each selected branch to assess the degree of general damage to the leaves and bark. Damage to the bark is difficult to measure due to the irregularities in boxwood bark, and the presence of epiphytic lichens, which complicate the observation and identification of bark damage. At the level of the new growth, the degree of general damage and length were measured to assess the condition and degree of recovery of the boxwood.

### Data analysis

The data for assessing the impact on boxwood (plot, plant, branch, and new growth) were collected at intervals and subsequently transformed into interval midpoints using the criterion shown in Supplementary material (Table S1). These values are percentages, which is why appropriate transformations of the variables were performed, converting the data to the arcsine of the variable, to stabilize the variance and approximating the data to a normal distribution. No transformations were made for the remaining variables. The raw data are publicly available at Figshare repository (Corominas, Vilar & Bou, 2026). For the statistical analysis of the data, R software (R Core Team, 2015) was used to analyse differences between treatments.

To analyse the condition of boxwood plants in the experimental plots, a generalized linear mixed model was applied for each scale variable: plot (Table S2), plant (Table S3), branch (Table S4), and new growth (Table S5). The treatments (T1 and T2) were established as fixed factors, along with the interaction between them. Additionally, to analyse seasonal variability, season was added as a fixed factor. The sampling plot and year were considered random factors (Tables S6, S7, S8 and S9), as the same plots were sampled periodically, making the measurements nonindependent (repeated measures). The assumptions of normality, homoscedasticity, and independence of residuals were evaluated for each model.

### Field permits

The treatment was applied by the *Servei de Sanitat Vegetal* of the *Departament d’Acció Climàtica, Alimentació i Agenda Rural* (*Generalitat de Catalunya*), the public authority responsible for pest management in Catalonia (Senmartí & Las Heras, 2020), under expedient code AG-2020-247. Monitoring of boxwood was conducted under fieldwork permits issued by *Generalitat de Catalunya* (expedient code AG-2020-1237).

## Results

### Plot

At the beginning of the monitoring study, there were already differences in boxwood coverage between control (74%), pheromones treatment (73%) and *B. thuringiensis* treatment (84%) plots. The pest was already present in the area and treatments were applied since a year ago. At the ending of the experiment, there were significant differences (*F*_2,18_ = 6.427, *p* < 0.01) in boxwood coverage (Fig. 2 and Table S2) between the treatment zones for treatment 1 (T1), where the boxwood coverage was greater in the areas treated with *B. thuringiensis*, with an average coverage value of 84%. In the control plots, a large part of the boxwood coverage (70%) consisted of necromass from highly defoliated boxwoods (71%).

**Figure 2:**
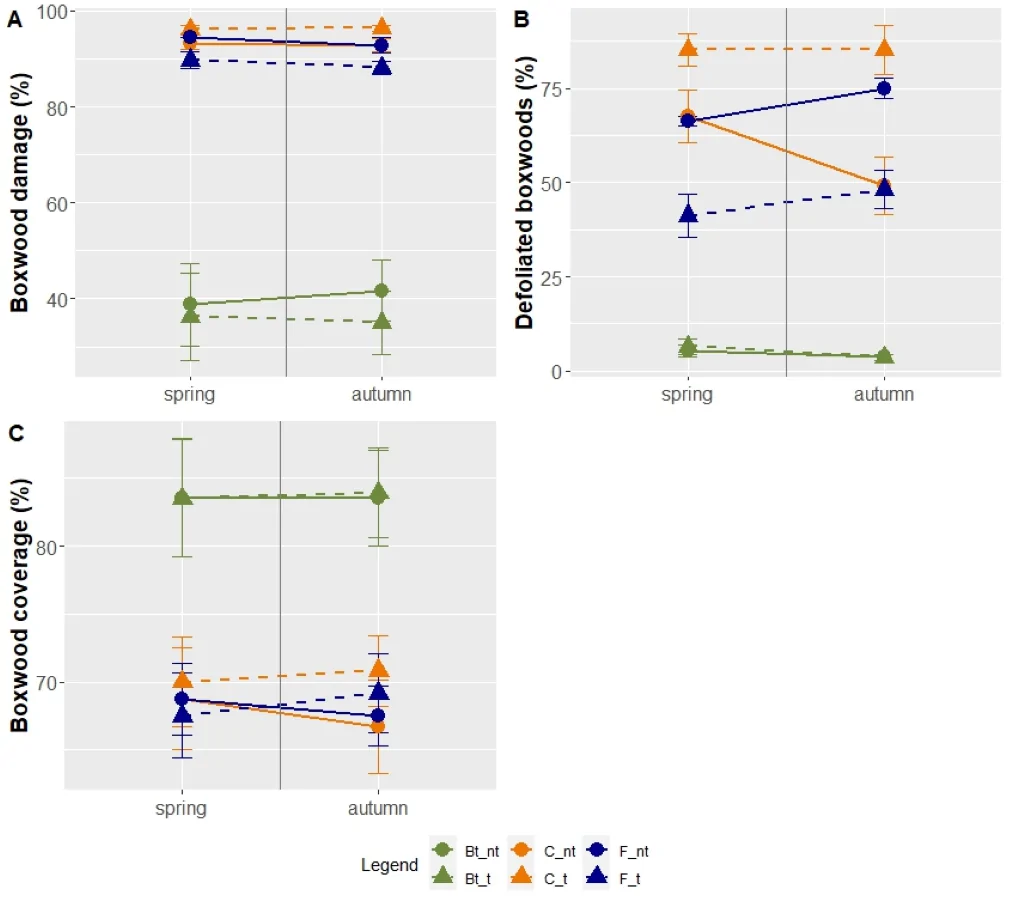
Variables measured at plot scale. Depending on the treatment received: (A) Degree of general damage (%); (B) Completely defoliated boxwoods (%); (C) Boxwood coverage (%). Treatment codes: Bt_nt (*Bacillus thuringiensis*), Bt_t (*Bacillus thuringiensis* combined with *Trichogramma*), C_nt (control), C_t (*Trichogramma*), F_nt (mass capture), F_t (mass capture combined with *Trichogramma*). The plot shows the mean value between the complete monitoring period (2020–2022). Error bars represent the standard error.

At the plot level, the degree of boxwood damage was analysed with two different variables: general damage (percent of defoliation) and completely defoliated boxwoods (percent of necromass individuals) (Fig. 2 and Table S2). In terms of general damage, a significant difference (*F*_2,18_ = 79.534, *p* < 0.001) was observed in treatment 1 (T1), with the plots treated with *B. thuringiensis* showed significantly less damage (40%) than did the control and mass trapping plots, which exhibited higher levels of general damage (94% and 92% respectively). The application of *Trichogramma* spp. (T2) did not have a significant effect on the general damage caused by the box tree moth, and differences among T2 groups were small. The seasonal variability of these values was very low, and no significant differences were observed between the spring and autumn campaigns.

Regarding completely defoliated boxwoods, significantly (*F*_2,18_ = 107.379, *p* < 0.001) lower values were observed in *B. thuringiensis* plots (4%) compared to those for the control (71%) and pheromone (58%) treatment groups (T1), but no significant differences were detected for the T2 treatment and seasonality. These results showed that treatment with *Trichogramma* spp. did not have a significant effect on this variable. Lower values of completely defoliated boxwoods were observed in plots combining parasitoids and pheromones than pheromones plots without parasitoids. In plots treated with parasitoids alone, values of completely defoliated boxwoods were not lower than those observed in the corresponding plots without parasitoids; in fact, values of completely defoliated plants were sometimes higher than those observed without parasitoids. In plots treated with *B. thuringiensis*, the percentage of completely defoliated boxwoods was already very low (close to 0%), with similar values in plots with *Trichogramma* spp. Despite the observed seasonal variability in this variable, no significant differences were detected.

The variability of plot variables associated with the different years of monitoring are very low.

### Individual boxwood plants

The damage to individual boxwood plants was assessed based on the impact on the individual plants, as well as the number of new shoots (Fig. 3 and Table S3). The results obtained for the degree of damage per plant showed a significant effect (*F*_2,18_ = 126.595, *p* < 0.001) of T1. Compared with the control treatment (97%), plants treated with *B. thuringiensis* showed the lowest damage (56%), whereas damage remained high under mass trapping (94%). No consistent pattern was observed for *Trichogramma* spp. across the treatments. There were no significant differences regarding the interaction between treatments or seasons.

**Figure 3:**
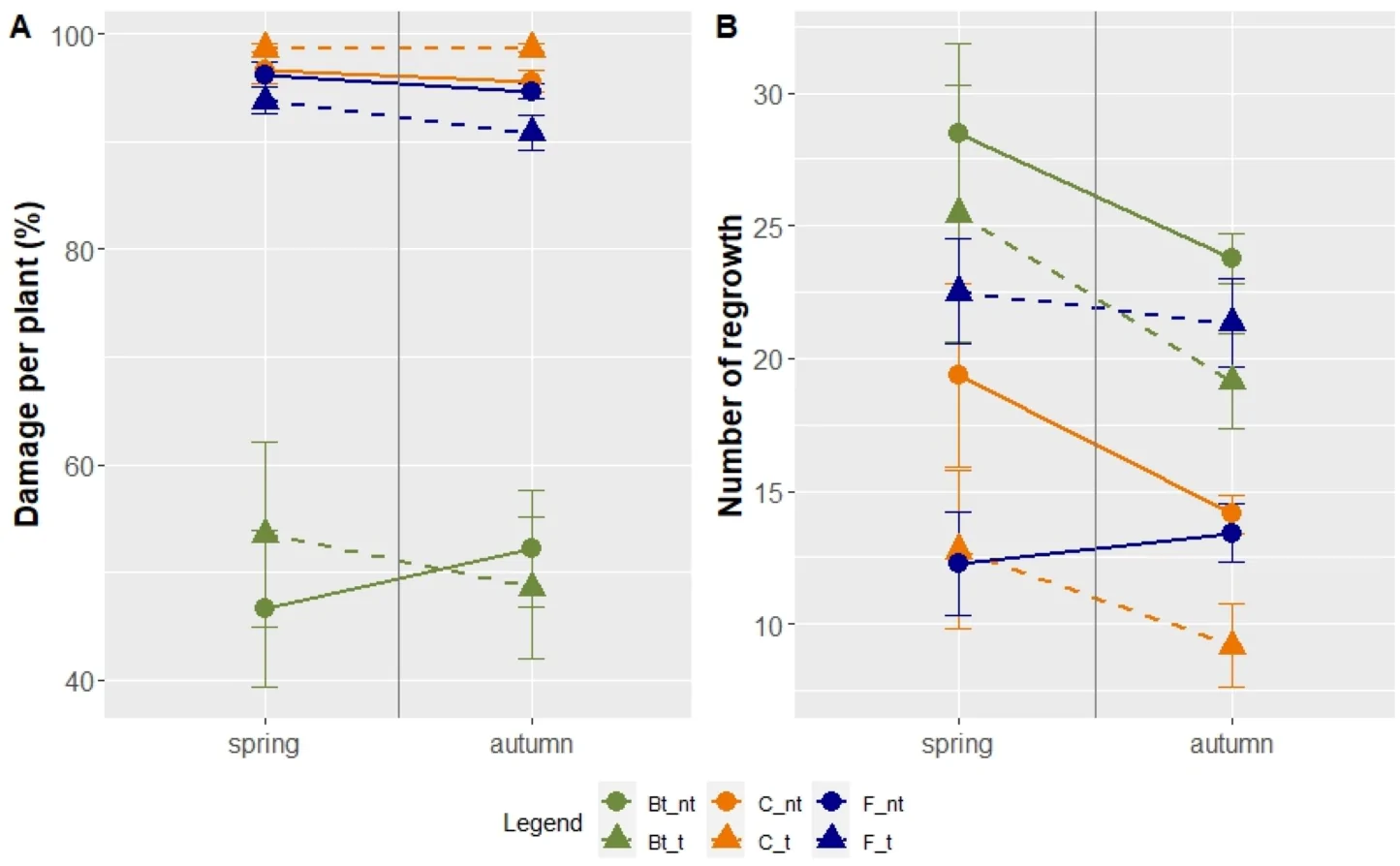
Variables measured on an individual boxwoods scale. Depending on the treatment received: (A) Damage per plant (%); (B) Number of sprouts (%). Treatment codes: Bt_nt (*Bacillus thuringiensis*), Bt_t (*Bacillus thuringiensis* combined with *Trichogramma*), C_nt (control), C_t (*Trichogramma*), F_nt (mass capture), F_t (mass capture combined with *Trichogramma*). The plot shows the mean value between the complete monitoring period (2020–2022). Error bars represent the standard error.

There were significant differences (*F*_2,18_ = 12.379, *p* < 0.001) in the number of new shoots between T1 treatments, and significant interaction between treatments and the season (*F*_2,18_ = 7.274, *p* < 0.01). The plots treated with *B. thuringiensis* had the greatest number of new shoots (29 regrowth on average), followed by those treated with mass trapping combined with parasitoids (22 regrowth on average). A significant interaction (*F*_2,18_ = 7.274, *p* < 0.01) between treatments T1 and T2 was detected for the number of new shoots. In the *B. thuringiensis* and the sole use of these parasitoids, lower values were observed compared to the corresponding plots without parasitoids. Conversely, in the mass trapping plots, higher values were observed in plots with *Trichogramma* spp. than in those without parasitoids. Regarding seasonal variability, the number of new shoots decreased in the autumn, except in some plots of the mass capture zone.

The variability of the individual boxwood variables among monitoring years was very low.

### Branches

On the branches, the overall damage to the bark and leaves was assessed, as the larva mainly defoliates the boxwood leaves but can also cause damage to the bark (Fig. 4 and Table S4). There were significant differences (*F*_2,18_ = 120.910, *p* < 0.001) in leaf damage between T1 treatments, with clearly lower values of damage in the treatments with *B. thuringiensis*. Seasonal differences were observed (*F*_2,18_ = 4.733, *p* < 0.05), with significant variability in damage during the spring campaign. The variability among years was very low.

**Figure 4:**
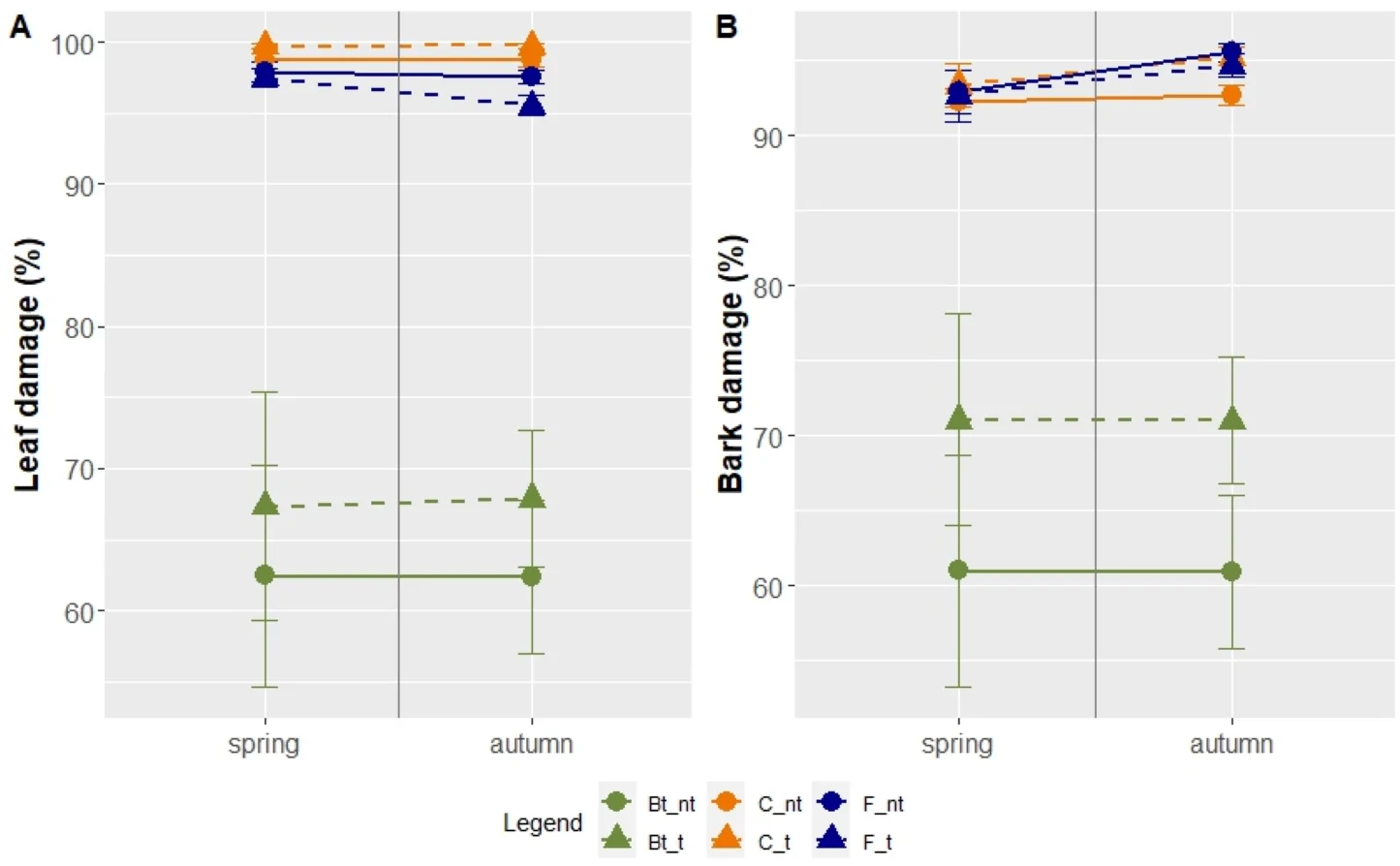
Variables measured at branch scale. Depending on the treatment received: (A) Leaf damage (%); (B) Bark damage (%). Treatment codes: Bt_nt (*Bacillus thuringiensis*), Bt_t (*Bacillus thuringiensis* combined with *Trichogramma*), C_nt (control), C_t (*Trichogramma*), F_nt (mass capture), F_t (mass capture combined with *Trichogramma*). The plot shows the mean value between the complete monitoring period (2020–2022). Error bars represent the standard error.

The difference in the average degree of bark damage was significant only for T1 (*F*_2,18_ = 85.500, *p* < 0.001), with lower values in the *B. thuringiensis* plots. A part of this variability is explained by the differences between years of monitoring.

### Regrowth

Regarding regrowth, the damage and length of the regenerated shoots were assessed (Fig. 5 and Table S5). For general damage, there were significant difference (*F*_2,18_ = 27.438, *p* < 0.001) between T1 treatments, as the degree of damage in the plots treated with *B. thuringiensis* (23%) and mass trapping (25%) were lower than that in the control (57%) plots. No significant differences were detected for T2 or for the interaction between factors. The season also had a significant effect (*F*_2,18_ = 4.781, *p* < 0.05), with a slight tendency towards a reduction in regrowth damage in some plots in the autumn. The variability among years was very low. A significant effect of T1 was detected for shoot length (*F*_2,18_ = 5.166, *p* < 0.05). Longer regrowth was observed in the *B. thuringiensis* plots, especially in the autumn. Variability among monitoring years was negligible.

**Figure 5:**
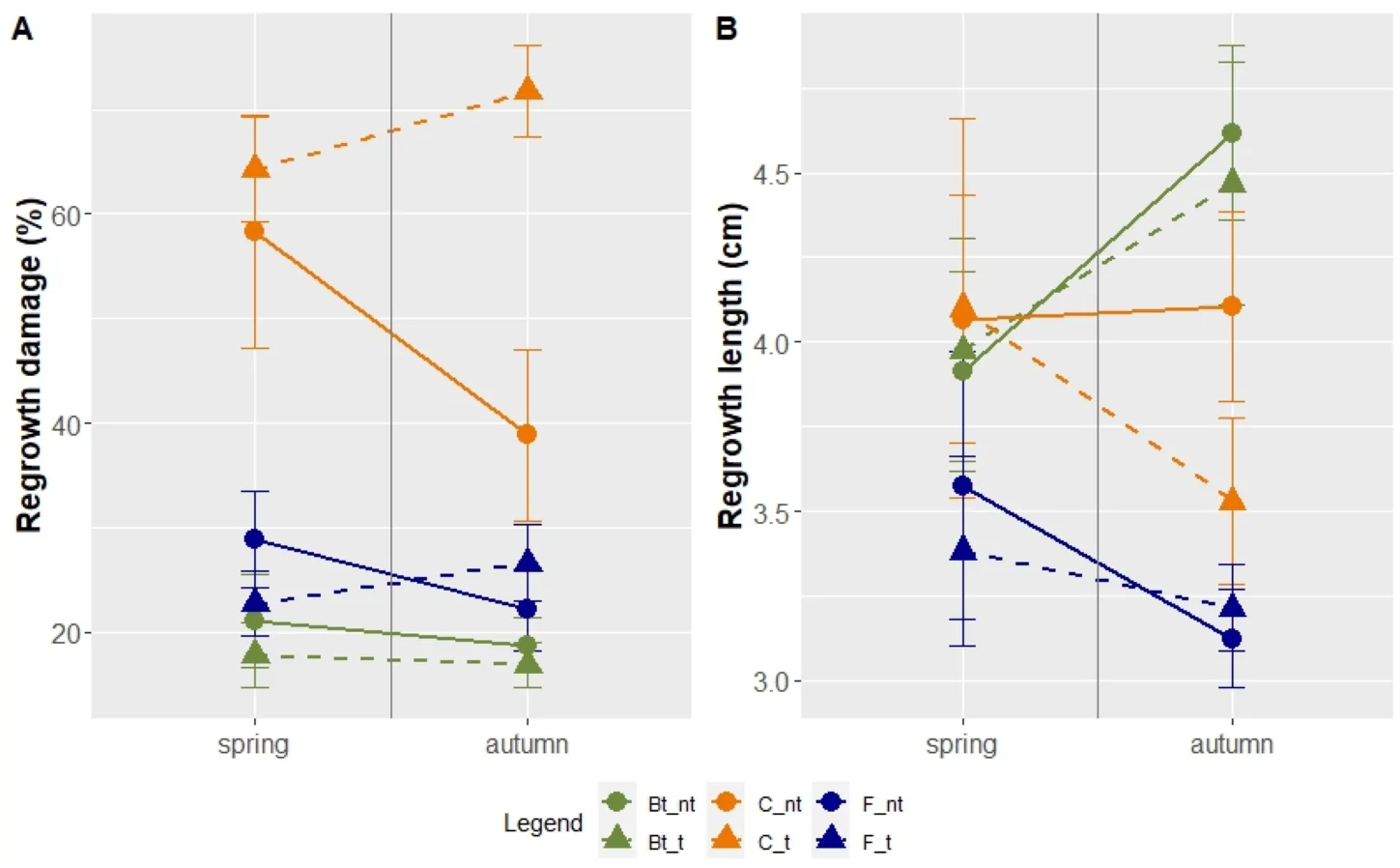
Variables measured on the regrowth scale. Depending on the treatment received: (A) Regrowth damage (%); (B) Regrowth length (cm). Treatment codes: Bt_nt (*Bacillus thuringiensis*), Bt_t (*Bacillus thuringiensis* combined with *Trichogramma*), C_nt (control), C_t (*Trichogramma*), F_nt (mass capture), F_t (mass capture combined with *Trichogramma*). The plot shows the mean value between the complete monitoring period (2020–2022). Error bars represent the standard error.

## Discussion

### The effectiveness of treatments

#### Phytosanitary treatment with Bacillus thuringiensis

The degree of general damage to boxwood at the plot and plant scale was lower in the *B. thuringiensis* treatment group than in the control and pheromone treatment groups, with very few plants being completely defoliated. The studied indicators demonstrate that treatment with *B. thuringiensis* reduces the degree of damage by protecting boxwood from defoliation while allowing spring growth. Branches treated with this pesticide suffer less damage, making it the most effective method among those tested. Additionally, it minimizes damage to new regrowth and is associated with greater regrowth during the period of greatest activity in the biological cycle of boxwood, with regrowth showing the greatest length in the autumn.

The effectiveness of this treatment was expected, as similar results had been obtained in other pest control trials using *B. thuringiensis* (Burjanadze, Supatashvili & Gorkturk, 2019). However, it is important to consider the limitations of applying *B. thuringiensis*, as boxwood bushes need to be sprayed with a cannon and pistol during the different periods corresponding to the emergence of the various generations of *C. perspectalis* for the treatment to be effective (Senmartí & Las Heras, 2020). Also, the study case is focused on a short-term study, but long-term studies are needed to evaluate if the resilience of *B. sempervirens* to defoliation decrease over long periods of time under high pest pressure. The impact of this product on the ecosystem should also be considered, as it affects all lepidopterans and may even affect some other insect species (Cawoy et al., 2011; Lacey et al., 2015). Furthermore, its continued use can lead to resistance and environmental contamination as it persists in the soil (Lambert & Peferoen, 1992). The effects on soil invertebrates and terrestrial ecosystems demonstrate that *B. thuringiensis* have a complex ecological footprint (Ragasruthi et al., 2024). Therefore, the use of this pesticide is currently regulated, and it can only be used as an insecticide in agriculture, gardening, or experimental trials (Regulation (EU) 540/2011).

#### Mass capture

The treatment effectiveness is quite different in the plots with mass trapping, as they exhibit high damage and maintain a similar trend to that of the control at the parcel, plant, and branch scales. Although a slight trend of decreasing leaf damage on the branches was observed, it was not distinguishable from the high damage of the control. These findings indicate that pheromone treatment is insufficient for preventing damage and mortality in these plants, showing limited effectiveness under high pest pressure. The obtained results are consistent with other studies in which pheromone treatments reduced the number of male adults but did not achieve satisfactory results for pest control (Van der Straten & Muus, 2010; Plant et al., 2019). It should be noted that mass trapping does not act directly on the stage of the box tree moth at which it causes damage, as it affects adults and not larvae. Therefore, a lesser effect on damage would be expected. In addition, this effect may not be detected due to the large number of adults flying over the plots (Senmartí & Las Heras, 2020), making traps insufficient. Furthermore, it should be considered that larvae can move a few metres (Artola et al., 2018), so limiting the number of adults laying eggs does not imply the absence of larvae later.

Nevertheless, compared with the application of *B. thuringiensis*, mass capture can reduce the impact of moths on shoots with a lower efficacy, but it does demonstrate a certain degree of effect compared to no treatment. This is relevant since shoots are a key element for assessing the recovery capacity of boxwood. The presence, growth, and preservation of shoots are crucial for the survival of boxwood in the context of high pest pressure.

The ability to preserve regrowth with mass capture suggests that this control method should not be ruled out. It is important to continue conducting experimental studies on this treatment and elucidate its optimal application (*e.g.*, Askari, Sefidkon & Allahverdi Mamaghani, 2023). In situations where pest pressure is continuous, it could be a valuable tool, allowing the reduction in mortality and the maintenance of shoots and promoting the survival of some individuals for future recovery of boxwood in a low-intensity pest situation. Moreover, its simple application could make it a preventive tool, providing rapid action against adults in some cases where pest pressure is low.

#### Parasitoids of the genus Trichogramma

The application of *Trichogramma* spp. parasitoids did not reduce damage to any of the studied variables, as the impact of the pest was high, as evidenced in the case of the control groups in the study. However, when applied in combination with *B. thuringiensis* and pheromones, this treatment appears to have a slight effect on reducing damage at the plant and plot levels. Nevertheless, at the branch and shoot levels, this effect was not observed, casting doubt on the positive effect of combining treatments. Thus, *Trichogramma* spp. application did not show clear effectiveness under the studied conditions for pest control in the natural environment. The effectiveness of its use in combination with other treatments remains uncertain since consistent results have not been obtained.

In fact, the limited effect detected for the use of parasitoids is comparable to the results observed in laboratory studies (Wan et al., 2014; Göttig & Herz, 2016a). Therefore, it would be expected that, in the long term, there would be no observed effectiveness in reducing damage to boxwood. Consequently, before applying these types of parasitoids in the natural environment, additional laboratory studies should be conducted to obtain high parasitism rates to confirm their effectiveness under stable and controlled conditions.

### Seasonal variability

In general, the seasonal variability was low in the conducted trial. Only a few indicators varied between the two sampling periods. For example, the coverage of boxwood did not change much between seasons; only some negative trends were observed in heavily affected plots, corresponding to the control or mass trapping treatments. This is, however, an expected long-term process due to boxwood defoliation, which causes necrosis in branches and trunks, eventually leading to breakage (obs. pers.) and a reduction in coverage in control and pheromone plots. This sustained situation over time may result in decreased coverage and increased boxwood mortality.

However, the shoots did show seasonal variation, as there was a tendency for a reduction in damage to spring shoots in the fall, especially in plots treated with *B. thuringiensis* and pheromones. It should be noted that this trend was also observed in untreated plots, as boxwood tends to regrow slightly once the last generation of caterpillars forms chrysalides, which are in good condition because the caterpillars have not defoliated them. The regrowth capacity of boxwood is noteworthy; despite damage accumulated from pests and summer water stress, it takes advantage of precipitation and relative humidity in September–October (Bou, Corominas & Vilar, 2021). The decreases of the pest in the colder months (Nacambo et al., 2014) and the seasonal dynamic of the regrowth, raise new insights to take into account on the resilience capacity of boxwood population. However, the recurrence of long-term damage is a limiting factor in this regrowth capacity. Therefore, it is necessary to analyse how boxwood populations respond to high levels of infestation or periods when pest pressure decreases, as the appearance of shoots in the fall can become a key element in the population dynamics of boxwood.

This seasonal variability is of interest in the current context of climate change (Hellmann et al., 2008), where an increase in temperatures and periods of drought is observed. These factors could lead to unfavourable conditions for the vitality of boxwood and its survival capacity. It is therefore relevant to continue long-term ecological monitoring (Larson et al., 2011) to analyse annual variability in broad temporal series.

## Conclusions

In terms of pest management, the results indicate that the absence of pest control measures for the box tree moth leads to massive defoliation, increased necromass, and mortality. To prevent this, treatment with *B. thuringiensis* appears to be the most effective method among those tested for pest control. However, there are several limitations to consider, such as its negative impact on the ecosystem and the difficulty of its application. Therefore, its use should be restricted to experimental trials or very specific cases of severe threats to the conservation status of boxwood at a regional scale. Mass trapping shows limited effectiveness as a general control tool because it has not been very effective in reducing the impact of boxwood. However, it should not be ruled out as a methodology for use in situations of high pest pressure to favour regrowth preservation and thereby avoid high boxwood mortality. Additionally, its use can also serve as a preventive measure when the species begins to be detected in a locality with low pest pressure. To improve the performance of this tool, more studies on trap density and its usage are necessary. The application of *Trichogramma* spp. did not show clear effectiveness under the studied conditions, and a higher success rate of parasitism in the laboratory would be needed before transferring this tool to the natural environment.

It is concluded that the currently available tools are insufficient for pest management, and an ecosystemic perspective needs to be incorporated to understand the impact of pests and their effects on biodiversity. In the context of this pest, it is important to consider future scenarios in which the pest continues to have a high impact. Specifically, changes in boxwood population trigger changes in species composition and ecosystem structure.

##  Supplemental Information

10.7717/peerj.21800/supp-1 Supplemental Information 1 Supplemental tables

## Additional Information and Declarations

### Data Availability

The following information was supplied regarding data availability: The data is available at figshare: - Corominas, Fiona; Vilar, Lluís; Bou, Jordi (2026). Data of: Which treatment is most effective at reducing the damage caused by the pest box tree moth? A case in a Mediterranean mountain. figshare. Dataset. https://doi.org/10.6084/m9.figshare.31576465.v1.
