# Supplementary material for: Which treatment is most effective at reducing the damage caused by the pest box tree moth? A case in a Mediterranean mountain
Source: PeerJ. 2026 Oct 22;14:e21800. doi: 10.7717/peerj.21800 (PMC13695964; doi:10.7717/peerj.21800)
Supplement: Supplementary file 1 [file peerj-14-21800-s001.docx]

**Supplementary material**

*Recommended citation:*

**Table S1.** Intervals used to assess the degree of affectation of the boxwood, and the values used in the statistical analysis.

**Table S2.** Results of the mixed generalized linear model (GLM) for each plot variable analysed.

**Table S3.** Results of the mixed GLM for each individual boxwood variable analysed.

**Table S4.** Results of the mixed GLM for each analysed branch variable.

**Table S5.** Results of the mixed GLM for each regrowth variable analysed.

**Table S6.** Random effects at plot scale.

**Table S7.** Random effects at individual boxwoods scale.

**Table S8.** Random effects at branches scale.

**Table S9.** Random effects at regrowth scale.

**Table S10.** Mean values for each treatment.

**Table S1.** Intervals used to assess the degree of affectation of the boxwood, and the values used in the statistical analysis.

| **Intervals of the affectation degree (%)** | **Values used in the analysis (%)** |
| --- | --- |
| 0 | 0 |
| 1-10 | 5,5 |
| 10-20 | 15 |
| 20-30 | 25 |
| 30-40 | 35 |
| 40-50 | 45 |
| 50-60 | 55 |
| 60-70 | 65 |
| 70-80 | 75 |
| 80-90 | 85 |
| 90-99 | 94,5 |
| 99 | 99 |
| 100 | 100 |

**Table S2.** Results of the mixed generalized linear model (GLM) for each plot variable analysed. For each fixed factor, the p value and Fischer F distribution are shown.

|  | | **T1** | | **T2** | | **T1:T2** | | **Season** | |
| --- | --- | --- | --- | --- | --- | --- | --- | --- | --- |
|  |  | **P** | **F_2,18_** | **P** | **F_1,18_** | **P** | **F_2,18_** | **P** | **F_1,95_** |
| **Plot** | **Boxwood damage** | <0.001 | 79.534 | 0.687 | 0.168 | 0.422 | 0.906 | 0.202 | 1.652 |
|  | **Defoliated boxwoods** | <0.001 | 107.379 | 0.532 | 0.405 | <0.001 | 13.189 | 0.347 | 0.893 |
|  | **Boxwood coverage** | <0.01 | 6.427 | 0.794 | 0.070 | 0.969 | 0.032 | 0.133 | 2.297 |

**Table S3.** Results of the mixed GLM for each individual boxwood variable analysed. For each fixed factor, the p value and Fischer F distribution are shown.

|  | | **T1** | | **T2** | | **T1:T2** | | **Season** | |
| --- | --- | --- | --- | --- | --- | --- | --- | --- | --- |
|  |  | **P** | **F_2,18_** | **P** | **F_1,18_** | **P** | **F_2,18_** | **P** | **F_1,95_** |
| **Individual boxwoods** | **Damage per plant** | <0.001 | 126.595 | 0.764 | 0.093 | 0.226 | 1.618 | 0.189 | 1.752 |
|  | **Number of regrowth** | <0.001 | 12.379 | 0.883 | 0.022 | <0.01 | 7.274 | <0.01 | 7.457 |

**Table S4.** Results of the mixed GLM for each analysed branch variable. For each fixed factor, the p value and Fischer F distribution are shown.

|  | | **T1** | | **T2** | | **T1:T2** | | **Season** | |
| --- | --- | --- | --- | --- | --- | --- | --- | --- | --- |
|  |  | **P** | **F_2,18_** | **P** | **F_1,18_** | **P** | **F_2,18_** | **P** | **F_1,95_** |
| **Branches** | **Leaf damage** | <0.001 | 120.910 | 0.298 | 1.150 | 0.259 | 1.457 | <0.05 | 4.733 |
|  | **Bark damage** | <0.001 | 85.500 | 0.077 | 3.519 | 0.161 | 2.023 | 0.529 | 0.400 |

**Table S5.** Results of the mixed GLM for each regrowth variable analysed. For each fixed factor, the p value and Fischer F distribution are shown.

|  | | **T1** | | **T2** | | **T1:T2** | | **Season** | |
| --- | --- | --- | --- | --- | --- | --- | --- | --- | --- |
|  |  | **P** | **F_2,18_** | **P** | **F_1,18_** | **P** | **F_2,18_** | **P** | **F_1,95_** |
| **Regrowth** | **Regrowth damage** | <0.001 | 27.438 | 0.125 | 2.595 | 0.061 | 3.278 | <0.05 | 4.781 |
|  | **Regrowth length** | <0.05 | 5.166 | 0.597 | 0.290 | 0.869 | 0.141 | 0.965 | 0.002 |

**Table S6.** Random effects of the mixed GLM for each variable at plot scale (variance and standard deviation).

|  | | **Plot** | | **Year** | | **Residual** | |
| --- | --- | --- | --- | --- | --- | --- | --- |
|  |  | **Variance** | **Std. Dev.** | **Variance** | **Std. Dev.** | **Variance** | **Std. Dev.** |
| **Plot** | **Boxwood damage** | 0.012 | 0.110 | 0.003 | 0.057 | 0.014 | 0.119 |
|  | **Defoliated boxwoods** | 0.011 | 0.106 | 0.008 | 0.088 | 0.022 | 0.149 |
|  | **Boxwood coverage** | 0.016 | 0.125 | 0.001 | 0.033 | 0.003 | 0.056 |

**Table S7.** Random effects of the mixed GLM for each variable at individual boxwoods scale (variance and standard deviation).

|  | | **Plot** | | **Year** | | **Residual** | |
| --- | --- | --- | --- | --- | --- | --- | --- |
|  |  | **Variance** | **Std. Dev.** | **Variance** | **Std. Dev.** | **Variance** | **Std. Dev.** |
| **Individual boxwoods** | **Damage per plant** | 0.004 | 0.065 | 0.002 | 0.041 | 0.016 | 0.127 |
|  | **Number of regrowth** | 10.185 | 3.191 | 3.418 | 1.849 | 35.200 | 5.933 |

**Table S8.** Random effects of the mixed GLM for each variable at branches scale (variance and standard deviation).

|  | | **Plot** | | **Year** | | **Residual** | |
| --- | --- | --- | --- | --- | --- | --- | --- |
|  |  | **Variance** | **Std. Dev.** | **Variance** | **Std. Dev.** | **Variance** | **Std. Dev.** |
| **Branches** | **Leaf damage** | 0.003 | 0.057 | 0.005 | 0.072 | 0.012 | 0.110 |
|  | **Bark damage** | 0.002 | 0.049 | 0.007 | 0.083 | 0.009 | 0.094 |

**Table S9.** Random effects of the mixed GLM for each variable at regrowth scale (variance and standard deviation).

|  | | **Plot** | | **Year** | | **Residual** | |
| --- | --- | --- | --- | --- | --- | --- | --- |
|  |  | **Variance** | **Std. Dev.** | **Variance** | **Std. Dev.** | **Variance** | **Std. Dev.** |
| **Regrowth** | **Regrowth damage** | 0.010 | 0.099 | 0.008 | 0.087 | 0.029 | 0.169 |
|  | **Regrowth length** | 0.260 | 0.510 | 0.000 | 0.000 | 0.716 | 0.846 |

**Table S10.** Mean values for each treatment.

|  | | **Season** | **Treatments** | | | | | | | | | | | | |
| --- | --- | --- | --- | --- | --- | --- | --- | --- | --- | --- | --- | --- | --- | --- | --- |
|  |  |  | **Bt_nt** | | **Bt_t** | | **C_nt** | | **C_t** | | **F_nt** | | **F_t** | | |
|  |  |  | **Mean** | **± SD** | **Mean** | **± SD** | **Mean** | **± SD** | **Mean** | **± SD** | **Mean** | **± SD** | **Mean** | **± SD** |  |
| **Plot** | **Boxwood damage (%)** | **Spring** | 47,500 | 23,629 | 52,500 | 26,300 | 92,125 | 4,750 | 94,500 | 0,000 | 94,500 | 0,000 | 94,500 | 0,000 |  |
|  |  | **Autumn** | 37,500 | 22,174 | 22,500 | 9,574 | 92,125 | 4,750 | 97,875 | 2,250 | 94,500 | 0,000 | 85,000 | 0,000 |  |
|  | **Defoliated boxwoods (%)** | **Spring** | 4,125 | 2,750 | 6,500 | 6,232 | 60,000 | 23,805 | 75,000 | 8,165 | 65,000 | 0,000 | 32,500 | 15,000 |  |
|  |  | **Autumn** | 2,750 | 3,175 | 2,750 | 3,175 | 52,500 | 27,538 | 95,625 | 2,250 | 80,000 | 5,774 | 52,500 | 12,583 |  |
|  | **Boxwood coverage (%)** | **Spring** | 84,750 | 13,907 | 82,250 | 14,722 | 72,500 | 12,583 | 70,000 | 10,000 | 70,000 | 10,000 | 67,500 | 9,574 |  |
|  |  | **Autumn** | 82,375 | 12,419 | 84,750 | 11,258 | 65,000 | 8,165 | 70,000 | 10,000 | 65,000 | 8,165 | 67,500 | 9,574 |  |
| **Individual boxwoods** | **Damage per plant (%)** | **Spring** | 57,797 | 14,162 | 75,672 | 6,380 | 95,188 | 4,300 | 97,844 | 0,732 | 97,313 | 1,261 | 95,563 | 1,706 |  |
|  |  | **Autumn** | 53,125 | 23,545 | 37,234 | 18,911 | 96,625 | 0,709 | 99,469 | 0,898 | 95,109 | 1,833 | 88,250 | 7,325 |  |
|  | **Number of regrowth** | **Spring** | 35,000 | 5,683 | 37,578 | 4,871 | 22,406 | 12,869 | 14,141 | 11,295 | 13,219 | 7,057 | 20,875 | 7,050 |  |
|  |  | **Autumn** | 25,156 | 2,289 | 19,688 | 3,452 | 14,281 | 1,606 | 6,547 | 5,685 | 13,875 | 3,105 | 23,359 | 6,163 |  |
| **Branches** | **Leaf damage (%)** | **Spring** | 78,150 | 7,613 | 87,909 | 5,839 | 98,247 | 2,059 | 99,350 | 0,649 | 98,313 | 1,036 | 98,294 | 0,344 |  |
|  |  | **Autumn** | 57,019 | 18,206 | 61,597 | 10,166 | 98,791 | 0,993 | 99,856 | 0,288 | 97,019 | 1,776 | 93,284 | 1,826 |  |
|  | **Bark damage (%)** | **Spring** | 76,859 | 4,986 | 88,253 | 3,351 | 94,203 | 1,244 | 94,390 | 1,733 | 95,738 | 0,694 | 96,284 | 0,447 |  |
|  |  | **Autumn** | 55,544 | 16,235 | 65,213 | 10,514 | 92,498 | 2,700 | 96,841 | 0,545 | 97,213 | 1,209 | 95,453 | 2,072 |  |
| **Regrowth** | **Regrowth damage (%)** | **Spring** | 31,900 | 6,811 | 23,978 | 4,454 | 78,988 | 13,406 | 57,235 | 16,692 | 36,449 | 11,870 | 24,198 | 8,799 |  |
|  |  | **Autumn** | 21,719 | 9,366 | 12,881 | 4,472 | 19,445 | 19,665 | 72,172 | 25,997 | 20,319 | 15,854 | 19,444 | 10,969 |  |
|  | **Regrowth length (cm)** | **Spring** | 4,231 | 0,732 | 3,486 | 0,604 | 4,458 | 1,349 | 4,610 | 1,755 | 3,793 | 1,332 | 3,408 | 0,677 |  |
|  |  | **Autumn** | 4,883 | 0,712 | 5,122 | 1,340 | 3,650 | 0,630 | 3,092 | 0,237 | 2,737 | 0,248 | 3,163 | 0,378 |  |
